# Diagnostic accuracy of initial chest radiograph compared to SARS-CoV-2 PCR in patients with suspected COVID-19

**DOI:** 10.1259/bjro.20200034

**Published:** 2020-08-05

**Authors:** Maria Tsakok, Robert Shaw, Andrew Murchison, Sarim Ather, Cheng Xie, Robert Watson, Andrew Brent, Monique Andersson, Rachel Benamore, Fiona MacLeod, Fergus Gleeson

**Affiliations:** 1 Department of Radiology, John Radcliffe Hospital, Oxford University Hospitals NHS Foundation Trust, Oxford, OX3 9DU, UK; 2 Department of Clinical Infection, John Radcliffe Hospital, Oxford University Hospitals NHS Foundation Trust, Oxford, OX3 9DU, UK; 3 MRC Weatherall Institute of Molecular Medicine, University of Oxford, John Radcliffe Hospital, Oxford, UK, OX3 9DU, UK; 4 Microbiology Laboratory, John Radcliffe Hospital, Oxford University Hospitals NHS Foundation Trust, Oxford, OX3 9DU, UK

## Abstract

**Objective:**

The chest radiograph (CXR) is the predominant imaging investigation being used to triage patients prior to either performing a SARS-CoV-2 polymerase chain reaction (PCR) test or a diagnostic CT scan, but there are limited studies that assess the diagnostic accuracy of CXRs in COVID-19.

To determine the accuracy of CXR diagnosis of COVID-19 compared with PCR in patients presenting with a clinical suspicion of COVID-19.

**Methods and materials:**

The CXR reports of 569 consecutive patients with a clinical suspicion of COVID-19 were reviewed, blinded to the PCR result and classified into the following categories: normal, indeterminate for COVID-19, classic/probable COVID-19, non-COVID-19 pathology, and not specified. Severity reporting and reporter expertise were documented. The subset of this cohort that had CXR and PCR within 3 days of each other were included for further analysis for diagnostic accuracy.

**Results:**

Classic/probable COVID-19 was reported in 29% (166/569) of the initial cohort. 67% (382/569) had PCR tests. 344 patients had CXR and PCR within 3 days of each other. Compared to PCR as the reference test, initial CXR had a 61% sensitivity and 76% specificity in the diagnosis of COVID-19.

**Conclusion:**

Initial CXR is useful as a triage tool with a sensitivity of 61% and specificity of 76% in the diagnosis of COVID-19 in a hospital setting.

**Advances in knowledge:**

.

Diagnostic accuracy does not differ significantly between specialist thoracic radiologists and general radiologists including trainees following training.

There was a 40% prevalence of PCR positive disease in the cohort of patients (*n* = 344) having CXR and PCR within 3 days of each other.

Classic/probable COVID-19 was reported in 29% of total cohort of patients presenting with clinical suspicion of COVID-19 (*n* = 569).

Initial CXR is useful as a triage tool with a sensitivity of 61% and specificity of 76% in the diagnosis of COVID-19 in a hospital setting

## Introduction

Initial studies have focused on CT findings and its diagnostic accuracy in COVID-19.^1–7^ CT has been demonstrated to be highly sensitive during its use as a first-line investigation for COVID-19 in mainland China where the outbreak commenced^3,4^, but there are very limited studies on the use of chest radiographs (CXR),^8–10^ and none that include patients with and without the disease presenting acutely to an emergency department. A study of 64 patients by Wong et al8, where the radiologists were asked to score whether the disease was present or absent, and its distribution and severity, demonstrated the initial CXR to have a 69% sensitivity for COVID-19 patients compared to 91% for initial SARS-CoV-2 reverse transcriptase polymerase chain reaction (PCR), in a cohort of 64 patients. However, the interpreting radiologists were aware that the entire cohort was PCR positive, and this may therefore represent an overestimate of the sensitivity of CXR. Meanwhile, PCR itself has a reported sensitivity of 70–80% in the diagnosis of COVID-19.^3,4^

The British Society of Thoracic Imaging (BSTI) have advised the use of the CXR as the first-line imaging investigation for patients presenting during the pandemic with a clinical suspicion of COVID-19 infection, with “immediate/hot” reporting of the CXR to differentiate classic/probable COVID-19 infection from non-COVID-19 infection. This result can be used to assist in the triage of patients awaiting diagnostic PCR tests.^11^ CT was to be reserved for cases with either diagnostic uncertainty or for patients that are significantly more hypoxic than would be expected from the CXR appearances. As an additional consideration, CT scanners must be decontaminated after patients with confirmed or suspected COVID-19 to reduce the infection risk to other acutely unwell or cancer patients. This takes time and has significant implications for service provision in the context of an already stretched CT capacity, further limiting the role that CT can play in the diagnosis of COVID-19 in many healthcare settings including the UK National Health Service.^12^

We reviewed the accuracy of CXR diagnosis and severity scoring of COVID-19 compared with PCR in patients presenting with a clinical suspicion of COVID-19. Additionally, as the CXRs are to be “hot” reported 24 h per day, we compared the diagnostic accuracy of thoracic radiologists to general radiologists including those in training, as they would also be “hot” reporting the chest radiographs.

In order to ensure that all reporters were adequately trained on reporting COVID-19 CXRs, a departmental tutorial took place and a dedicated training set of CXRs with notes and references was made available for all reporters.

## Methods and materials

**Figure 1.**
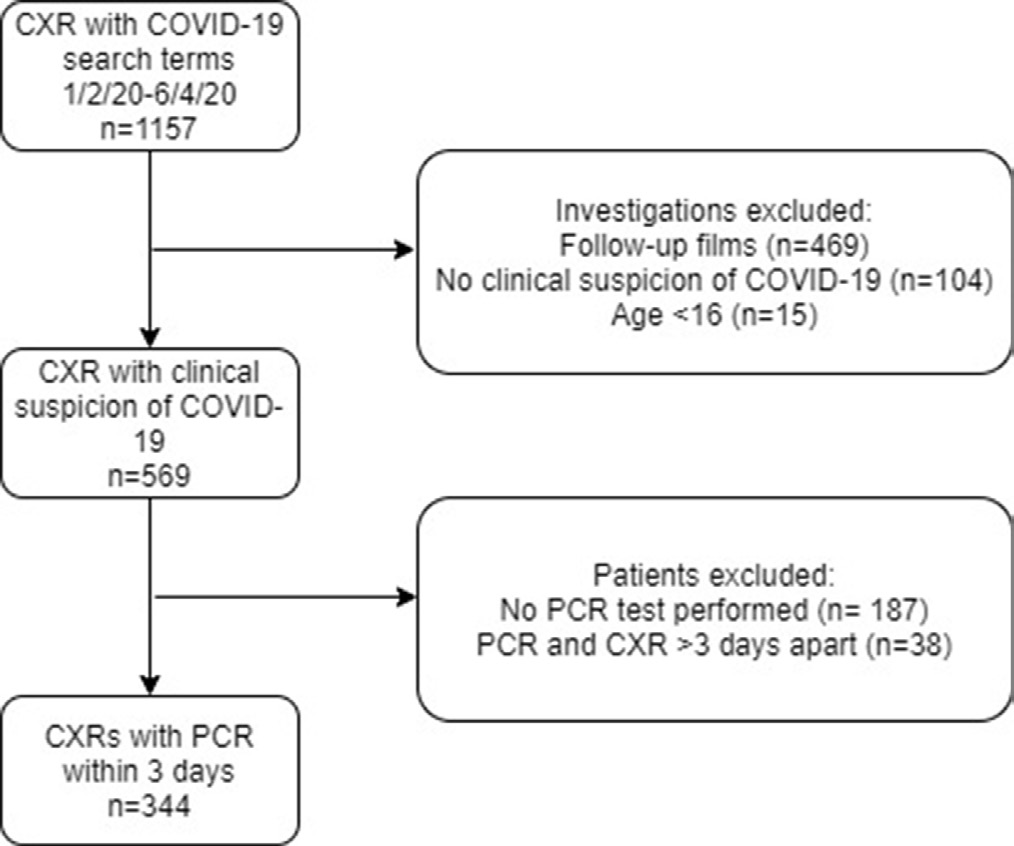
Investigations flow chart

**Figure 2.**
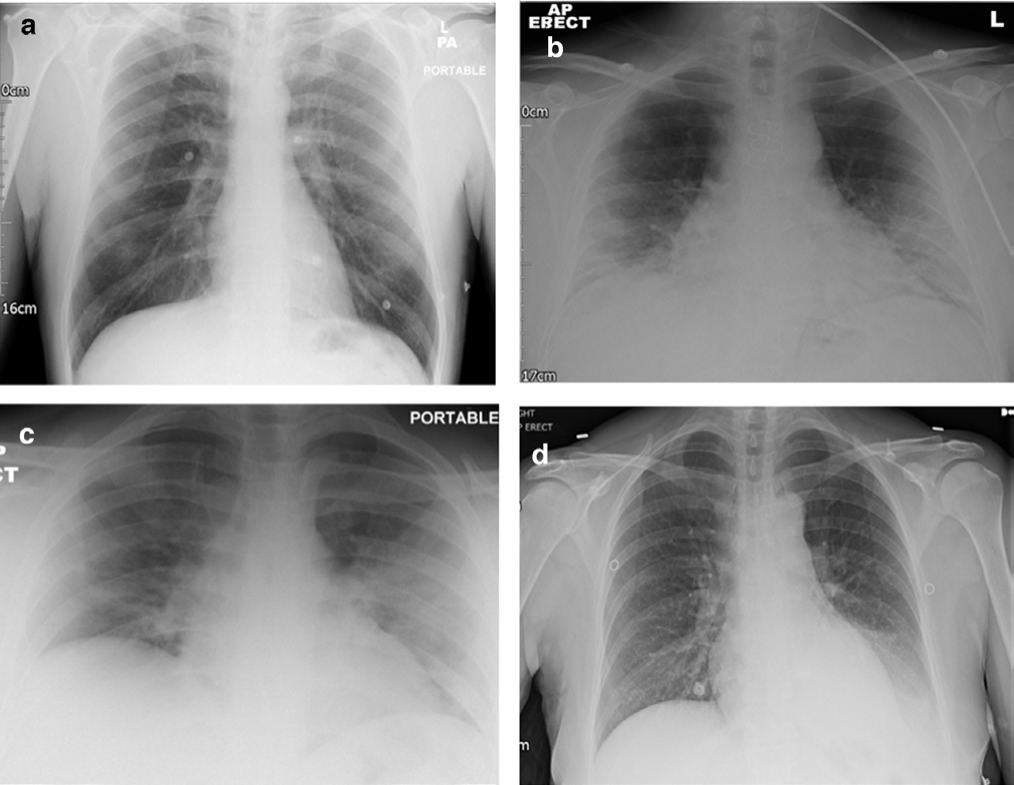
Example chest radiographs for the report categories. *A* = normal, *B* = indeterminate, *C* = classic/probable COVID-19, *D* = non-COVID-19

We performed an electronic search of the Radiology Information System (RIS) to identify all consecutive CXRs between 1 February and 6 April 2020 performed due to clinical suspicion of COVID-19. The comprehensive list of COVID-19 related terms is provided (Supplementary Table 1) . Exclusion of follow-up imaging, those without a clinical suspicion of COVID-19 (according to the CXR request and electronic patient record) and children resulted in a total of 569 patients for inclusion in the study (see investigation flowchart, Figure 1). Symptomatic data were collected (HC, AL, SAP, SP, IS, RS) from the electronic patient record. CXR reports were reviewed, blinded to the PCR result (MT, AM), and classified into the following categories: normal, indeterminate for COVID-19, classic/probable COVID-19, non-COVID-19 pathology and not specified (Figure 2 for example images). Severity reporting into mild, moderate and severe or whether not specified, and reporter expertise (thoracic radiologists *vs* general radiologist including trainees) were documented.The project received institutional approval as a service improvement audit and informed consent was waived.

### PCR testing

At this point, early in the pandemic, the PCR testing strategy was to test only those who had a clinical suspicion of COVID-19, who also had a severe enough syndrome requiring hospital admission. Those with suspected mild disease were discharged without PCR testing. A combined nose and throat swab were taken using a flocked swab and transported to the laboratory in viral transport medium. Guidance on taking a good quality swab was provided through the hospital website. RNA extraction was carried out on the Qia Symphony platform and amplification of SARS-CoV-2 RNA was carried out on the Qiagen Rotorgene using the RdRp assay.^13^

## Results

### Patient and investigation characteristics

569 consecutive patients were identified from the clinical history within the CXR requests and subsequently the electronic patient record, as having a clinical suspicion of COVID-19. The median age was 61 years (range 17–104 years), with a male:female ratio of 51:49. Of the 569 patients, thoracic radiologists reported 28%. 67% (382/569) had PCR tests, with 18% (104/569) having multiple tests (Table 1). Median turnaround time of PCR test from collection to result was 27 h compared to 7 h for the CXR report. The most common symptoms were cough, fever and shortness of breath.

**Table 1.** Patient characteristics. Percentages in parentheses

Characteristics	Results
Age (years)	
Median age	61
Age range	17–104
16–29	55 (9)
30–49	126 (22)
50–69	176 (31)
>70	212 (37)
Male:female ratio	51:49:00
Median time intervalfrom CXR report to RT-PCR result	28 h
Median time interval from symptom onset to CXR date	5 days
RT-PCR	
Positive	137 (24)
Negative	245 (43)
Not tested	187 (33)
Symptoms	
Cough	400 (70)
Fever	360 (63)
SOB	356 (63)
Chest pain	153 (27)
Sputum	120 (21)
Fatigue	112 (20)
Nausea and vomiting	100 (18)
Myalgia	95 (17)
Diarrhoea	77 (14)
Headache	67 (12)
Confusion	59 (10)
Sore throat	58 (10)
Rigors	44 (8)
Coryza	43 (8)
Sweats	42 (7)
Anosmia	20 (4)
Haemoptysis	12 (2)
Rash	6 (1)
Arthralgia	5 (<1)
Conjunctivitis	0 (0)
Tests	
Total	382 (67)
Number of tests	
1	278 (49)
2	59 (10)
3	24 (4)
4	12 (2)
5	3 (<1)
6	4 (<1)
7	2 (<1)

### CXR reporting

**Figure 3.**
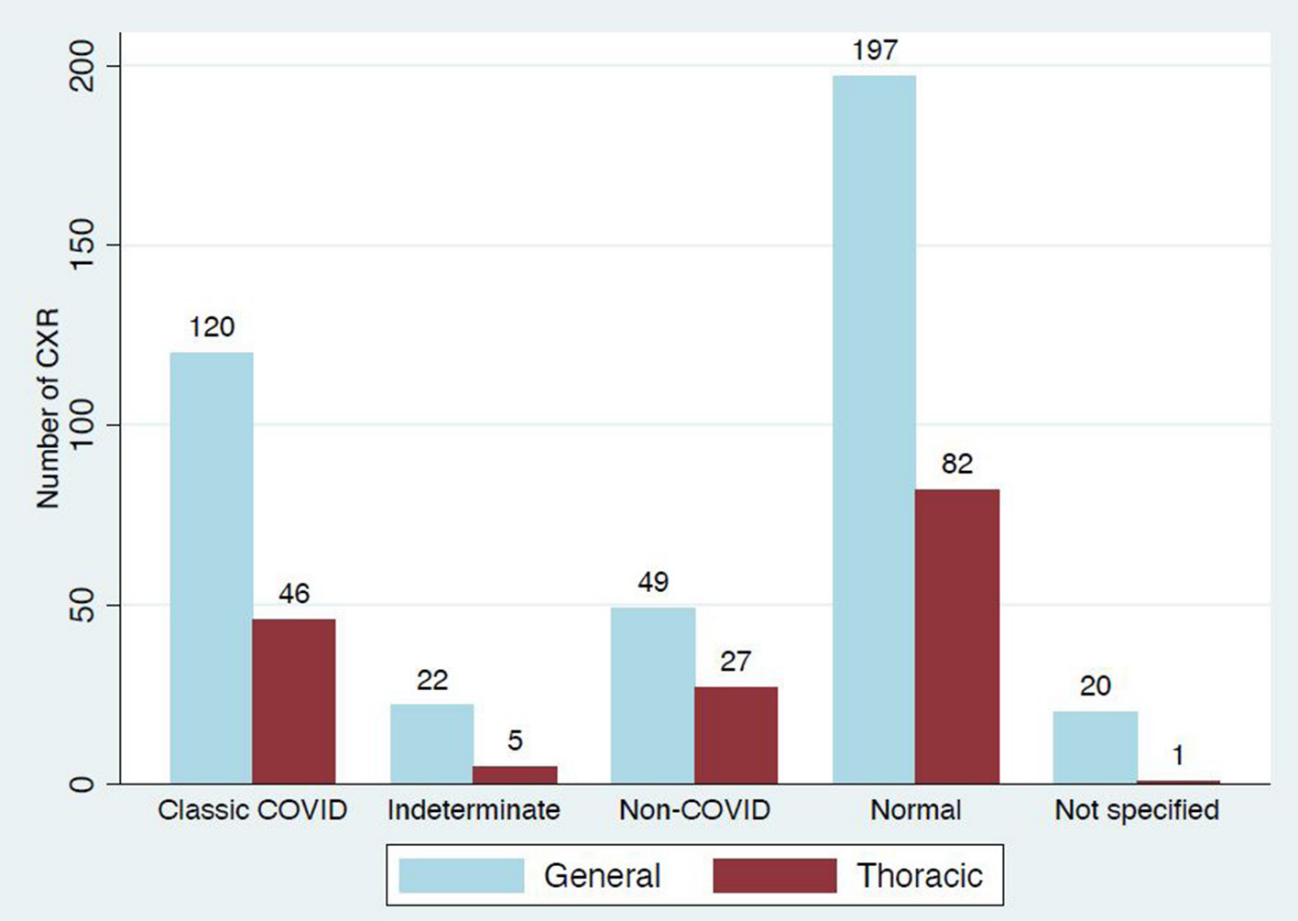
Case numbers by report category and reporter type

**Figure 4.**
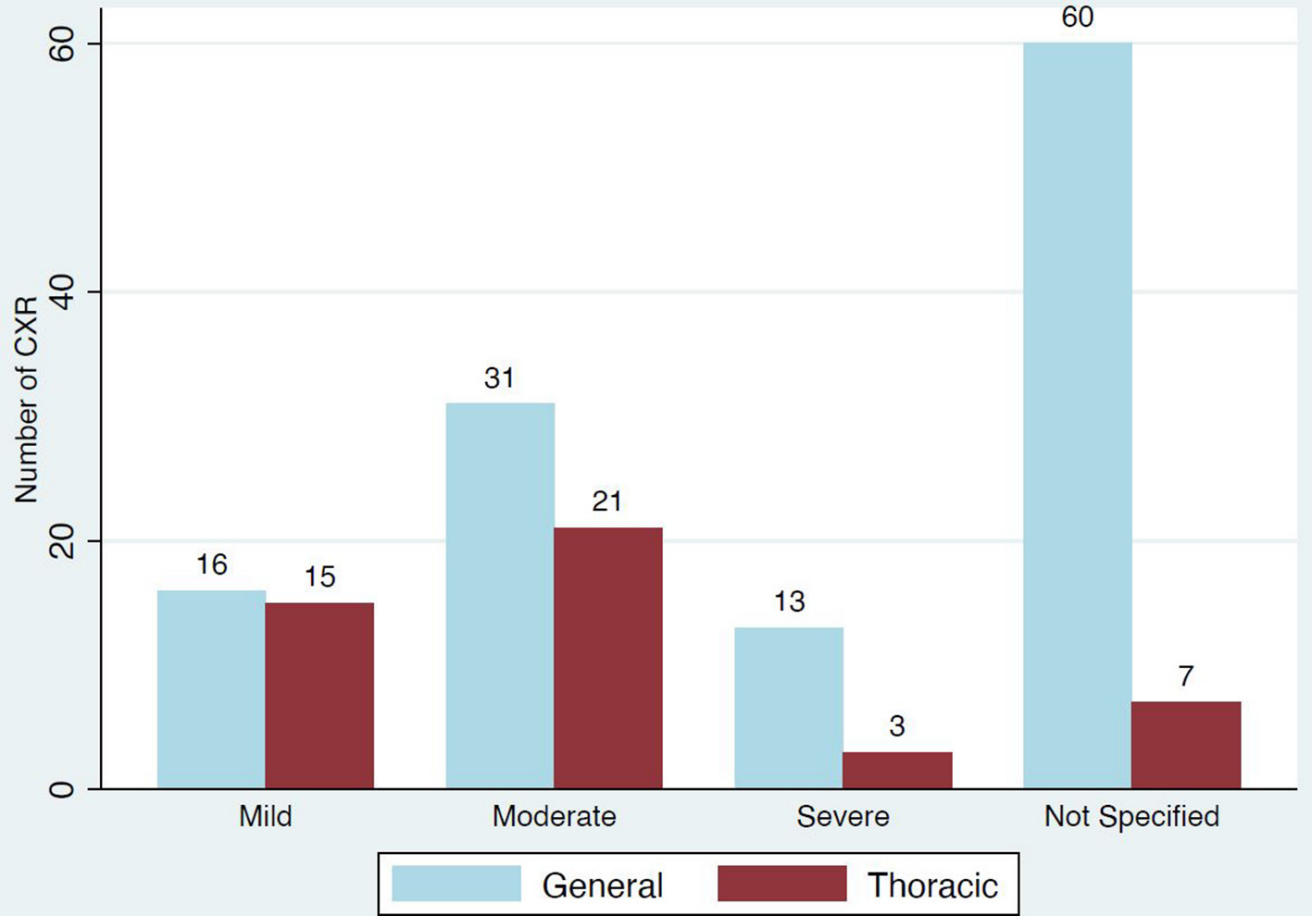
Reporting of severity in classic/probable COVID-19.

Classic/probable COVID-19 was reported in 29% (166/569) of the initial cohort (Figure 3). Of the 60% (99/166) that reported severity, 31 were mild, 52 moderate and 16 severe (Figure 4).

### PCR testing

**Figure 5.**
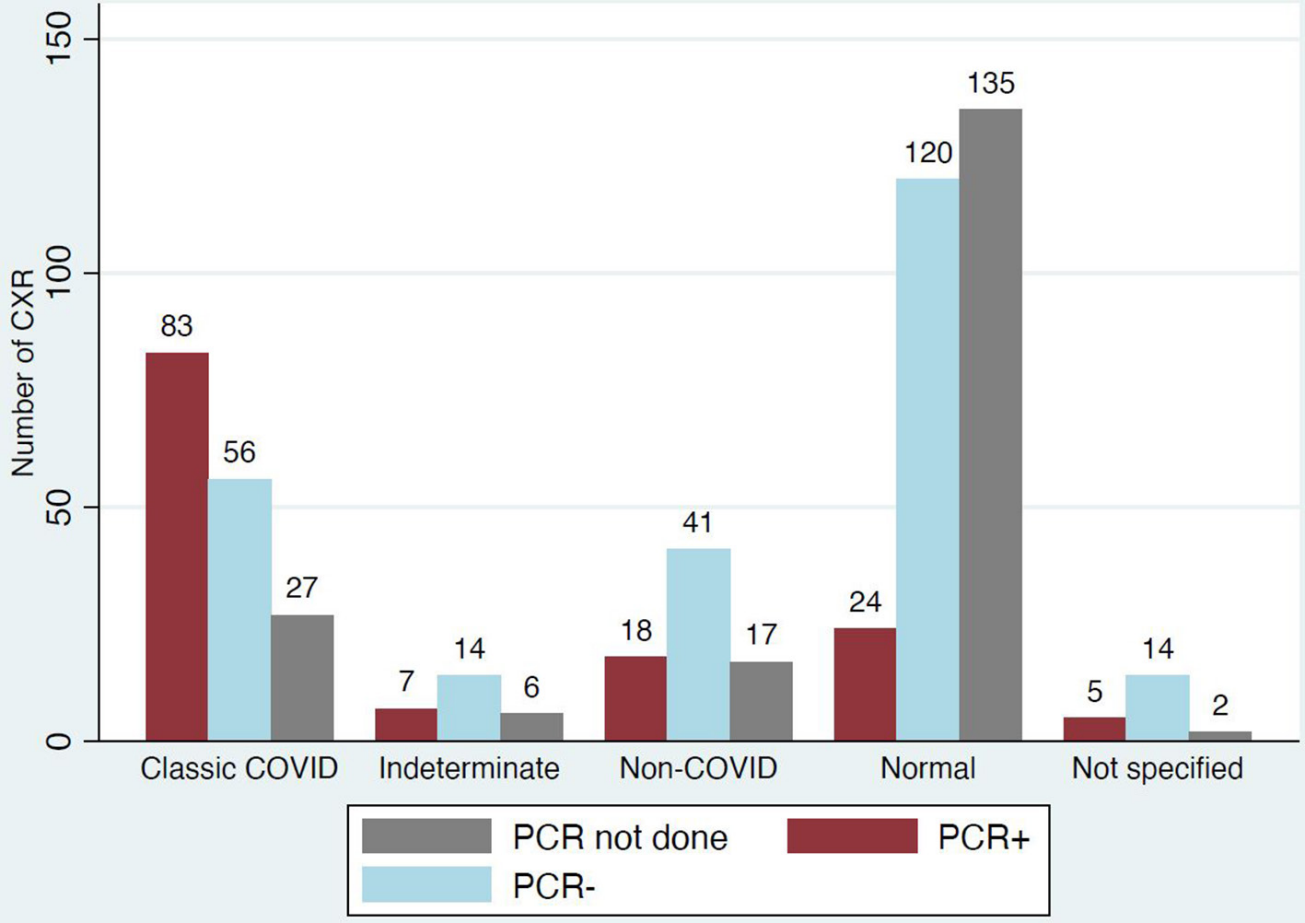
PCR testing as per report category

PCR testing per report category is shown in Figure 5. PCR testing was conducted in 84% (139/166) of those with CXRs reported as classic/probable COVID-19 and was positive in 60% of cases (83/139), while in patient with CXRs reported as normal PCR testing took place in 52% (144/279) and was positive in only 17% (24/144).

### CXR correlation with PCR

In the subset of patients with PCR testing within 3 days of CXR, classic/probable COVID was reported in 38% (132/344) of cases, and there was a 40% (137/344) prevalence of PCR positive disease. 4% (24/569) of patients became positive after the initial swab.

When compared to PCR as the reference standard, CXR had a 61% sensitivity (95% CI 52–69%) and a specificity of 76% (95% CI 70–82%) (Tables 2 and 3).

**Table 2.** 2 × 2 table of CXR (classic/probable COVID) *vs* PCR

	PCR positive	PCR negative
**CXR positive** (*Classic/probable COVID*)	83	54
**CXR negative**	49	158

**Table 3.** Diagnostic accuracy parameters of CXR (classic/probable COVID) *vs* PCR

Statistic	Value	95% CI
Sensitivity	61%	52–69%
Specificity	76%	70–82%
Positive Likelihood Ratio	2.6	1.9–3.4
Negative Likelihood Ratio	0.52	0.41–0.64
Disease prevalence	40%	35–45%
Positive Predictive Value	63%	54–71%
Negative Predictive Value	75%	68–80%

26% (87/344) cases with paired PCR result were reported by thoracic radiologists. The chest radiologists’ sensitivity of 63% (95% CI 44–79%) and specificity 76% (95% CI 63–87%) did not differ from non-thoracic radiologists 60% (95% CI 50–69%) and specificity of 76% (69–83%) by Fisher’s exact test. The addition of the indeterminate category resulted in a slight increase in sensitivity and a slight decrease in specificity as would be expected (Tables 4 and 5); however, this was not found to be statistically significant, by Fisher’s exact test.

**Table 4.** 2 × 2 table of CXR (classic/probable COVID or indeterminate) *vs* PCR

	PCR positive	PCR negative
**CXR positive** (*classic/probable or indeterminate for COVID*)	90	62
**CXR negative**	47	145

**Table 5.** Diagnostic accuracy parameters of CXR (classic/probable COVID or indeterminate) *vs* PCR

Statistic	Value	95% CI
Sensitivity	66%	57–74%
Specificity	70%	63–76%
Positive Likelihood Ratio	2.2	1.7–2.8
Negative Likelihood Ratio	0.49	0.38–0.63
Disease prevalence	40%	35–45%
Positive Predictive Value	59%	51–67%
Negative Predictive Value	76%	69–81%

18% (104/569) had two or more PCR tests. Out of 81 patients who had more than one PCR, but whose initial PCR test was negative and within 3 days of their CXR, 24 subsequently tested positive. Nine of these patients had a diagnosis of classic/probable COVID-19 on initial CXR, a median of 6.1 days prior to the positive PCR report.

## Discussion

Our study demonstrates the diagnostic accuracy of initial CXR compared to PCR in a SARS-CoV-2 cohort of otherwise unselected patients presenting acutely to the emergency department. By including a consecutive sample of all patients being investigated for COVID-19 with a CXR, our results are likely to be generalisable to the presenting case load in other similar settings.

UK testing strategy at this point in the pandemic only included patients with severe enough illness to require admission to hospital, the cohort with paired CXR and SARS-CoV-2 PCR will therefore be biased towards those with more severe disease. Another important consideration is that the sensitivity of both PCR and CXR is highly dependent on the timing within the disease course. To minimise bias arising from test timing, we limited our analysis to cases in which CXR and PCR had been performed within 3 days.

An important limitation of the study is the use of upper respiratory tract SARS-CoV-2 PCR as the reference standard against which to compare the diagnostic performance of CXR. The sensitivity of PCR is affected by the timing of sampling, the quality sample taken as well as the performance of the assay used. Published studies suggest upper respiratory tract PCR has a sensitivity of 70–80%^3,4^ against a composite reference standard incorporating clinical, radiological and microbiology data. We chose not to use a composite reference standard including a clinical data and/or CT as a consensus definition is lacking. However, the imperfect sensitivity of PCR does mean that some PCR negative cases may have been COVID-19 cases. Our estimate of the specificity of CXR should therefore be treated with caution and may be an underestimate.

According to reporting guidelines for our institution, use of the indeterminate category for reporting films with a clinical suspicion of COVID-19 was limited to reduce the burden of further CT imaging and allow more decisive triage. We have demonstrated in further analysis that addition of the indeterminate report category to equate to CXR diagnosis of COVID-19 increases sensitivity but reduces specificity.

An overall sensitivity of 61% for the CXR is comparable to the report by Wong et al8, which only included patients with PCR positive COVID-19 disease. The sensitivity is higher than reported by Weinstock et al,^9^ who reported that 58.3% of PCR positive patients presenting with mild symptoms to a non-emergency department in New York City had a normal CXR, a setting with a markedly different testing strategy.

Importantly, because the CXR appearances of COVID-19 are now relatively well reported, it is possible to produce training sets for general radiologists and those in training that enable them to practice for this diagnosis to the same standard as specialist radiologists. It may also be possible to train emergency doctors to the same level of competence.

In summary, we have shown that among patients presenting to an emergency department, CXR is 60% sensitive and 76% specific compared to upper respiratory tract SARS-CoV-2 PCR; and that once trained, all radiologists may report the CXR with similar levels of accuracy.
